# Assessment of signal quality measured with a smart 12‐lead ECG acquisition T‐shirt

**DOI:** 10.1111/anec.12682

**Published:** 2019-07-24

**Authors:** David Fouassier, Xavier Roy, Anne Blanchard, Jean‐Sébastien Hulot

**Affiliations:** ^1^ Assistance Publique Hôpitaux de Paris, Centre d'Investigations Cliniques CIC1418 Hôpital Européen Georges Pompidou Paris France; ^2^ Paris Cardiovascular Research Center PARCC Inserm UMR970 Paris France

**Keywords:** cardiology, ECG, e‐textile, garment, Holter, telemedicine, wearable

## Abstract

**Background:**

Ambulatory ECG monitoring is typically achieved using portable devices with limited number of surface leads, autonomy, and length of recording. Smart garments with multiple conductive textile electrodes provide great promise to perform continuous and comfortable ECG monitoring.

**Methods:**

We evaluated the ECG signal quality measured on healthy subjects with a smart 12‐lead ECG acquisition T‐shirt or a 12‐lead Holter recording. ECG signals were recorded during 3 min with both techniques in three resting positions (supine, seated, standing) and while walking. Three readers independently assessed ECG patterns and evaluated the denoising of the isoelectric line, the distinction of p waves, R peaks and RR intervals, and the possible appreciation of cardiac rhythm in at least 3 leads.

**Results:**

Thirty healthy subjects (70% males, 29.5 ± 7.8 years) were enrolled in the study. For all three resting conditions, cardiac rhythm was appreciated in 100% of recordings with distinction of p waves, R peaks, and isoelectric line in >97% of recordings. Appreciation of cardiac rhythm was lower in the walking conditions with both techniques (53.3% vs. 46.7%, Holter vs. smart T‐shirt, *p* = .60) mainly due to difficulties to distinguish p waves. These results were consistent across both genders. All ECG parameters (heart rate, PR, QRS and QTC intervals) were comparable between both techniques. No skin irritation was seen with the textile electrodes.

**Conclusions:**

A smart T‐shirt with 13 textiles electrodes allows short‐duration 12‐lead ECG acquisition with quality levels comparable to Holter recordings. The novel device should now be evaluated for long‐term non‐invasive ECG monitoring.

## 1 INTRODUCTION

The diagnosis of most cardiac arrhythmias is challenging because of their short‐lasting, paroxysmal, and sometimes asymptomatic nature. Typically, ambulatory devices such as Holter monitors are proposed in patients presenting with a suspicion of cardiac rhythm disorders in order to capture ECG on short periods (e.g., typically 24 hr). Standard devices have a limited autonomy and consequently have a limited sensitivity to detect arrhythmias (Healey & Wong, 2019; Seet, Friedman, & Rabinstein, 2011). Recent studies have shown that prolonged ECG monitoring up to 4 weeks improves the detection of cardiac arrhythmias such as paroxysmal atrial fibrillation by a factor of more than five (Gladstone et al., 2014). Similarly, the yield of short‐term Holter monitoring in patients with syncope may be as low as 1%–2% thus urging the recent guidelines to rather recommend the use of longer‐term ECG monitoring devices (Brignole et al., 2018). In addition, most devices have a limited number of leads (e.g., 1–3) which can be sufficient to capture and interpret the cardiac rhythm. A higher number of recording leads (up to 12‐leads as in a standard ECG), however, offer a greater value to localize cardiac arrhythmias, to monitor complex cardiac arrhythmias, or to analyze cardiac repolarization.

During the last years, a number of new devices have been designed and proposed for continuous non‐invasive ECG monitoring. A first category of portable devices measures a surrogate of cardiac activity (e.g., pulse rate with photoplethysmography or recorders with inner electrodes) to identify irregular beats as a potential marker of cardiac arrhythmias and then send the information through wireless transmitters such as smartphones (Bruce et al., 2016; Gropler, Dalal, Hare, & Silva, 2018; Tabing, Harrell, Romero, & Francisco, 2017; William et al., 2018). These devices have been demonstrated to provide good quality single‐lead ECG signal and have good accuracy to detect pulse irregularities (Taggar, Coleman, Lewis, Heneghan, & Jones, 2016). As they rely on commercially available consumer devices, they would thus appear as particularly suitable for population‐based screening of arrhythmias (Haberman et al., 2015; Turakhia et al., 2019). A second category of devices uses conductive textile electrodes that are inserted in a garment thus providing great promise to perform continuous and comfortable ECG monitoring in everyday life. Single‐lead ECG monitoring with textile electrodes was recently reported as providing high signal quality for adequate rhythm analysis (Balsam et al., 2018; Pagola et al., 2018; Steinberg et al., 2019; Tsukada et al., 2019).

We report the evaluation of ECG signal quality measured on healthy subjects with a novel smart 12‐lead ECG acquisition T‐shirt as compared to a 12‐lead Holter recording.

## 2 METHODS

### 2.1 Study design

We performed a prospective, monocentric, open‐label study in healthy subjects (clinicaltrials.gov NCT 03725462). To be enrolled, subjects had to be older than 18 years and without any personal history of cardiovascular disorders. All participants underwent ECG monitoring wearing the 12‐lead smart T‐shirt (Cardioskin™) or a 12‐lead Holter (Spiderview, Sorin) in four different positions: supine rest, seated rest, standing rest, and while walking. For each recording session, subjects firstly wear the smart T‐shirt and recordings were acquired during 3 min for each position (lying, sitting, standing, walking). After finishing the complete recording session, a technician removed the smart T‐shirt and examined the body of the subject to eliminate undesirable effects on the skin. Gel electrodes were then placed on the subject and positioned as for a standard 12‐lead ECG. The electrodes were connected to the Holter recorder. ECG recording was then acquired during 3 min for each position (lying, sitting, standing, walking) as previously performed with the smart T‐shirt.

The study was conducted in accordance with Guidelines for Good Clinical Practice and Declaration of Helsinki and approved by relevant local ethic committee (CPP Sud‐Est II, 2017‐A00648‐45). All enrolled subjects provided informed consent to study participation.

### 2.2 Smart ECG acquisition T‐shirt

**Figure 1:**
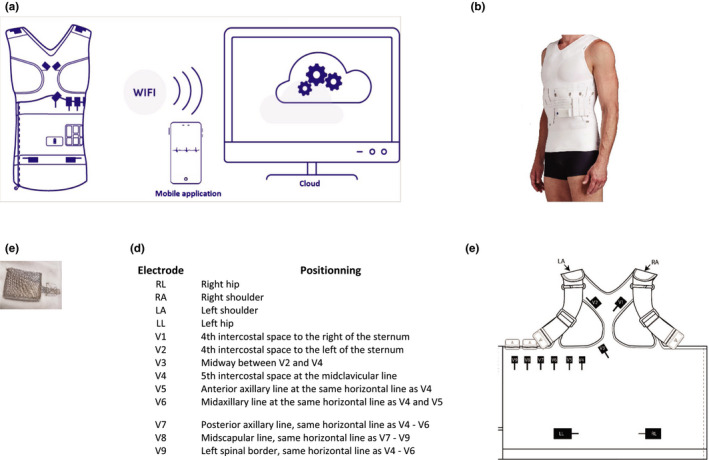
(a) Schematic representation of the platform composed of a T‐shirt with textile electrodes, a battery, and a recorder including a software package to receive and transmit wirelessly the signal. (b) T‐shirt constituted with 13 textiles electrodes, a removable battery, and a recorder. (c) electrode made of silver yarns and of hydrogel pads. (d) positions of the 13 electrodes integrated into the T‐shirt. (e) Map of the electrodes positions in the T‐shirt (opened for representation)

Cardioskin™ is a smart garment constituted with 13 textiles electrodes for ECG recording, a battery and a wireless recorder, built under the international standard IEC 60601‐2‐47. The electrodes and wires are fully embedded in the garment and positioned as defined by 15‐lead standard ECG (Figure 1a,b). The electrodes are made of silver yarns and of hydrogel pads that release water vapor in order to moisture the skin to get a better electrophysiological signal (Figure 1c‐e). This smart T‐shirt is a CE‐marked medical device that can thus monitor ECG on 15 leads, which represent a higher number than offered by the comparator (e.g., 12‐lead Holter recording). For this reason, the ECG recording with the smart T‐shirt was limited to 12 leads by deactivating ECG signal capture on posterior leads (corresponding to V7, V8, V9 leads on a standard ECG). The smart T‐shirt sizes were selected according to the underbust size (i.e., the measure around the chest just below breast).

### 2.3 Evaluation of ECG signal quality

For both techniques and for each position, the high pass and low pass filters used for ECG signal processing were respectively 0.05 Hz and 25 Hz for Holter and 0.04 Hz and 51.58 Hz for the smart T‐shirt. ECG tracings of 10 s were extracted at random and then assessed for quality by three independent cardiologists. Each expert used a pre‐specified binary questionnaire to first evaluate three criteria regarding ECG patterns as follows:

- Is the isoelectric line stable and denoised? Yes/ no
- Are the p waves visible? Yes/ no
- Are the R peaks visible and the RR intervals measurable? Yes/ no

Then, for every recording, each expert was asked to state on the diagnostic quality defined as the possible appreciation of cardiac rhythm in at least 3 leads. The individual scores were collected and the final ratings correspond to the agreement of at least two evaluators. ECG parameters (i.e., Heart rate, PR, QRS, and QT intervals) were measured on all available recordings in all positions. QT was corrected for heart rate using the Bazett's formula.

### 2.4 Statistical analysis

Categorical variables are presented as percentages and were compared between groups using chi‐square or Fisher's exact test as appropriate. Continuous variables are expressed as mean ± *SD* and were compared with a Mann–Whitney *U* test. Statistical analyses were performed using Prism v7 (Graphpad software Inc).

## 3 RESULTS

A total of 30 healthy subjects (21 males, 9 females) were enrolled in the study, and ECG recordings were available for all participants, for both techniques and for all positions. The mean age was 29.5 ± 7.8 years and was not significantly different between genders (30.2 ± 8.5 years for men vs. 27.8 ± 6.2 years for women, *p* = .39). The mean underbust size was 91.2 ± 13.6 cm, ranging from 67 cm to 124 cm, corresponding to anticipated sizes in a healthy subject population.

**Figure 2:**
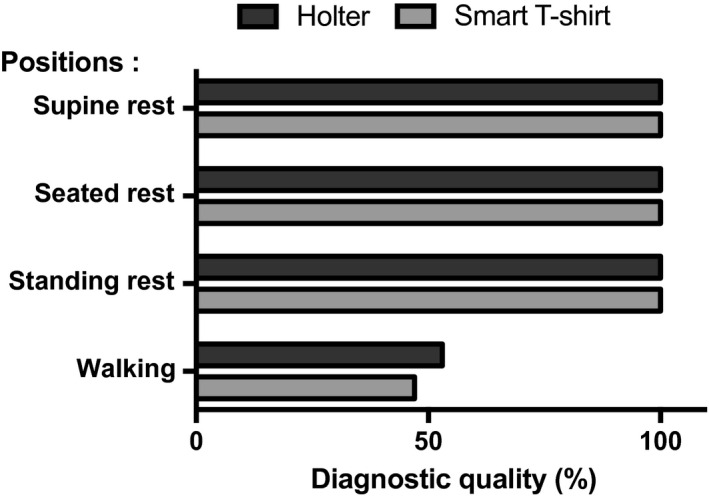
Diagnostic quality (%) defined as the distinction of a sinus rhythm in the four studied positions obtained with the smart T‐shirt and with Holter ECG

**Figure 3:**
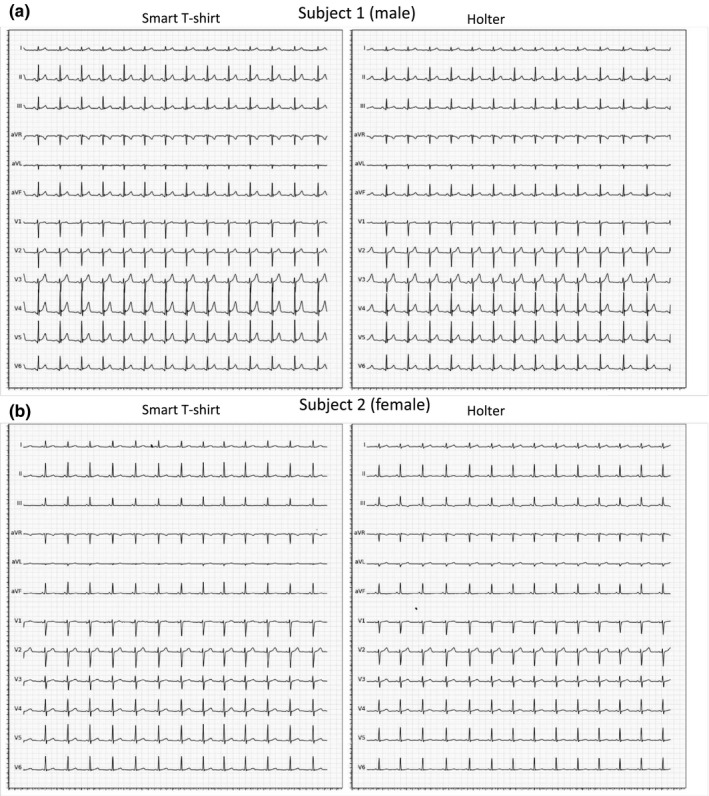
Representative ECG tracings with the smart T‐shirt and with Holter ECG obtained in standing position in (a) a male subject and (b) a female subject

For all three resting conditions, the distinction of a sinus rhythm was possible for all recording obtained with both techniques (Figure 2). The distinction of p waves, R peaks, and R interval was seen on all ECG tracings with both techniques. The isoelectric line was found stable and correctly denoised on 99.9% ECG records performed with the smart T‐shirt, and on 96.7% of the Holter recordings (*p* = NS). Figure 3 shows representative ECG signal obtained with the smart T‐shirt in standing rest in two different subjects (male and female).

**Figure 4:**
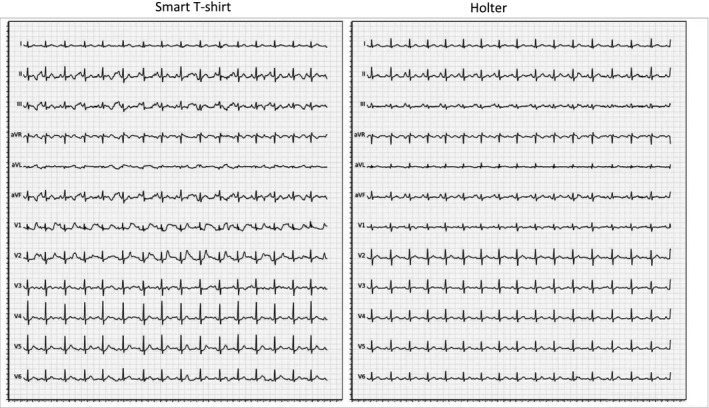
Representative ECG tracings obtained with the smart T‐shirt and with Holter ECG during walking in the same subject

For both techniques, the quality of ECG patterns as well as the overall ECG signal quality was lower in walking conditions. Exercise was notably associated with higher difficulties to distinguish p waves with both techniques (46.7 ± 50.7% with smart T‐shirt vs. 53.3 ± 50.7% with Holter, *p* = .60). Under these conditions, the smart T‐shirt was associated with statistically significant lower rates of finding a stable and denoised isoelectric line (6.7 ± 25.4% with smart T‐shirt vs. 36.7 ± 49.0% with Holter, *p* = .01), but despite this higher noise, the R peaks and RR intervals were detectable in 86.7 ± 34.6% with smart T‐shirt versus 83.3 ± 37.9% with Holter (*p* = .71). Overall, difficulties in identifying sinus rhythm on the ECG strips were largely driven by the difficulties to detect p waves, and the final appreciation of cardiac rhythm in walking conditions was comparable between both techniques (46.7 ± 50.7% with smart T‐shirt vs. 53.3 ± 50.7% with Holter, *p* = .60). Figure 4 shows a representative ECG recording obtained with both methods during walking.

We further looked to the agreement between both techniques (Table 1) and found that, under walking conditions, there was no significant advantage for one technique over the other. Both techniques concordantly provided low‐ or high‐quality scores for 18 subjects, while ECG recordings for the 12 other subjects were randomly better with one or the other technique (5 vs. 7 for smart T‐shirt vs. Holter, *p* = .26).

**Table 1:** Diagnostic quality according to ECG recording techniques under walking conditions

Diagnostic quality		Holter	
		Low	High
Smart T‐shirt	Low	9	7
	High	5	9

ECG parameters were measured and compared between both techniques according to the recording positions (Table 2). All measures obtained with the smart T‐shirt were highly comparable to the ones measured with Holter, and none of the parameters were significantly different between both techniques.

**Table 2:** ECG parameters

	Supine rest		Seated rest		Standing rest		Walking	
	Smart T‐shirt	Holter	Smart T‐shirt	Holter	Smart T‐shirt	Holter	Smart T‐shirt	Holter
Heart rate (bpm)	70.4 ± 11.5	70.6 ± 11.4	72.4 ± 11.9	73.3 ± 12.7	85.6 ± 15.2	84.4 ± 16.0	88.5 ± 11.4	91.2 ± 12.9
PR (ms)	147.5 ± 16.4	149.1 ± 18.6	144.1 ± 20.5	145.7 ± 21.2	137.2 ± 20.2	138.1 ± 19.1	136.3 ± 18.5	141.2 ± 18.2
QRS (ms)	78.4 ± 5.6	77.4 ± 5.8	78.1 ± 4.9	77.7 ± 5.0	76.5 ± 6.2	76.3 ± 5.7	76.0 ± 5.7	78.0 ± 4.4
QTc (ms)	382.6 ± 21.7	382.4 ± 23.8	389.6 ± 22.9	388.2 ± 21.6	389.9 ± 20.5	388.1 ± 22.2	398.3 ± 18.5	391.6 ± 19.6

Note:

Values are presented as mean ± *SD*. *N* = 30 per group except for walking conditions (*n* = 14 for the smart T‐shirt and *n* = 16 for Holter). All statistical comparisons were non‐significant.

ECG recordings with the smart T‐shirt or with the Holter were perfectly tolerated. No skin irritation was seen with the textile electrodes.

## 4 DISCUSSION

Since their first report, smart textiles are considered strategic to reduce morbidity and health care costs associated with cardiovascular disorders (Lymberis & Olsson, 2003). Indeed, smart garments can integrate multiple conductive textile electrodes and provide great promise to perform continuous and comfortable ECG monitoring. We report the first results of a novel smart T‐shirt (Cardioskin™) that integrates a high number of electrodes to provide a 12‐lead ECG recording. The electrodes were positioned in the T‐shirt in order to mimic the typical pattern obtained with a standard 12‐lead ECG machine. This pilot study thus establishes the feasibility of generating such a device for ECG monitoring and shows that the signal quality and measures obtained with the smart T‐shirt were comparable to Holter recordings in the 4 different studied positions. The signal quality was remarkably excellent in all resting conditions, indicating the efficiency of textiles electrodes to conduct ECG signal, their correct positioning and the overall ability of this smart T‐shirt to perform a wireless ECG recording. We notably did not observe any important differences between genders. In addition, all measured parameters were comparable to the ones obtained with the holter (Table 2), indicating that monitoring of main ECG parameters (notably the length of QT interval) is feasible with the smart T‐shirt.

Using a smart garment may thus represent a relevant solution to perform long‐term ECG recordings while avoiding the usual Holter limitations. Holter monitors are ambulatory devices used to capture ECG on short periods (typically 1–2 days). They are small and portable but require the use of skin electrodes connected with long cables, which are perceived as uncomfortable and prone to misconnections. In addition, Holter represents a relative discomfort that may limit patients in their daily activities. Prolonged monitoring with conventional Holter systems is also limited by potential skin reaction (Tu, Spence, Kalman, & Davis, 2014). The smart T‐shirt we studied here could thus represent a potential significant advance for ECG recordings by combining quality ECG signals and comfortable wireless and wearable ECG recorder.

The signal quality was significantly decreased during exercise, however, with both techniques. As electrodes were different with both techniques, the similar decrease in quality was more likely due to body movements and capture of skeletal muscle activity. Our study thus shows that ECG recording during exercise is more complicated. Consequently, an increase in comfortability with smart garments could also lead to an increase in patient's activities in their daily life thereby impacting the overall quality of recording. This issue deserves further investigations.

Another solution for long‐term ECG monitoring relies on the subcutaneous implantation of insertable cardiac monitors. These subcutaneous devices can perform ECG recording for up to 3 years with a low rate of noise burden (Reinsch et al., 2018). These devices have been studied and proved useful in selected cases of unexplained syncope and palpitations, as well as in atrial fibrillation management (Giancaterino, Lupercio, Nishimura, & Hsu, 2018). Smart garments, such as the one studied here, could appear as a non‐invasive alternative on the future but the relative indications of both types of devices remain to be determined.

As a pilot study, we first assessed ECG signal quality in short captions (e.g., 3 min) and whether using a smart T‐shirt is an appropriate solution to enable long‐term ECG recordings now remain to be demonstrated. In addition, we here performed a study in healthy subjects with normal sinus rhythm. The yield of the smart T‐shirt to detect and diagnose cardiac arrhythmias in patients also remains to be demonstrated. Other devices have recently been tested to detect frequent arrhythmias such as atrial fibrillation in a large number of patients (Pagola et al., 2018; Tabing et al., 2017; Turakhia et al., 2019; William et al., 2018). However, none of these devices have achieved routine clinical use for the ECG recording. In addition, and to the best of our knowledge, none of these solutions provide a full‐ECG recording as proposed by this smart T‐shirt. Finally, the smart T‐shirt is made in a flexible textile so that it can adapt to different morphologies. The ECG signal quality was here assessed in healthy subjects, and further studies are required to evaluate the smart T‐shirt in subjects with extreme body shapes.

In conclusion, a smart T‐shirt constituted with 13 textiles electrodes allows short‐duration 12‐lead ECG acquisition with quality levels comparable to Holter recordings. This novel device should now be evaluated for long‐term non‐invasive ECG monitoring.

## CONFLICT OF INTERESTS

No disclosures relevant to the submitted work. Outside the submitted work: JSH has received personal fees and non‐financial support from Novartis, personal fees from Amgen, personal fees from BMS, personal fees and non‐financial support from Servier. JSH is supported by the French National Research Agency (ANR‐17‐CE17‐0015‐02 and ANR‐18‐CE14‐0032‐02), Fondation Leducq (grant 18CVD05), Fédération Française de Cardiologie, France Génomique, Era‐CVD (ANR‐16‐ECVD‐0011‐03, Clarify project) and is coordinating a French PIA project (2018‐PSPC‐07, PACIFIC).
